# Basic proposal for evaluation of plant genetic resources to generate new crops

**DOI:** 10.3389/fpls.2025.1507521

**Published:** 2025-02-19

**Authors:** I. Darío Flores-Sánchez, Manuel Sandoval-Villa, Libia Iris Trejo-Téllez

**Affiliations:** Department of Edaphology, Colegio de Postgraduados, Montecillo, Texcoco, Mexico

**Keywords:** underutilized species, methodology, plant genetic resources, evaluation, food diversity

## Abstract

Given the reduced diversity of foods available in production systems, a factor linked to malnutrition in society, it is necessary to evaluate new plant genetic resources for human consumption. Underutilized or abandoned plant species, wild, semi-domesticated or domesticated, are an alternative to this problem. However, the lack of skills in people interested in this species, and the little attention paid to these resources in research centers, leads to a lack of basic data on characterization and evaluation, and makes it difficult to identify germplasm with potential for improvement purposes or for direct use. The objective of the proposal is to raise a basic theme to characterize and evaluate plant genetic resources in greenhouses and hydroponics, to propose and generate alternative crops with topics such as seed germination, traits of agronomic interest, nutrient absorption, phenology, fruit quality and secondary metabolites, which serves as a methodological guide, and meets the recommendations of the World Health Organization (WHO) and the Food and Agricultural Organization of the United Nations (FAO), on the need to generate data for the use of the biodiversity of underutilized or abandoned species, which will allow to increase the diversity of foods with important nutrimental content for the population.

## 1 Introduction

Worldwide, a reduced diversity of foods is reported in production systems; aspect linked to the problem of malnutrition in the population, resulting from an inadequate intake of nutrients, minerals and vitamins for the development and maintenance of a healthy body (Li et al., 2020). Currently, 1 in 3 people is affected by at least one form of malnutrition, such as nutrient deficiency (FAO and WHO, 2019).

The reduced diversity is due, among other factors, to the low use of plant species utilized to satisfy food needs, since of the 5,538 species that have been used by some method of use, 103 provide 90% of the calories consumed and corn, rice, wheat, soybeans and potatoes are the crops that contribute 60% of these calories (Li et al., 2018; N’Danikou and Tchokponhoue, 2019).

This situation has left aside species with important nutrient content and beneficial components, such as underutilized or abandoned species, which could help address the problem of malnutrition, as mentioned by the World Health Organization (WHO, 2020), by contributing to a healthier, balanced and diverse diet, ensuring an adequate supply of nutrients (Li et al., 2020). This need is also raised in the second objective of the Sustainable Development Goals relating to improving the nutrition of the population (ONU, 2014).

However, two of the problems that limit their study, characterization and evaluation for their use are that they have been marginalized in research centers, as they are not included in basic crops, and the reduced skills of people interested in these resources to address the challenges of interdisciplinary research, a fundamental aspect when studying new genetic resources of wild or semi-domesticated plants. This condition is exacerbated by the lack of a methodology to conduct the study and selection of germplasm (Padulosi et al., 2013; Nixwell et al., 2022).

For the above reasons, the following thematic proposal is presented, which is considered basic in the evaluation of plant genetic resources to propose and generate alternative crops, to serve as a guide for people interested in the study of these resources.

## 2 Background

### 2.1 Underutilized or abandoned species

These species are considered an alternative to address malnutrition problems due to their important nutritional content of proteins, vitamins, micronutrients and nutraceutical compounds, poorly supplied by the main basic crops rich in carbohydrates (FAO, 2018; Li et al., 2020).

They belong to a broad group of wild, semi-domesticated and domesticated plants (Padulosi et al., 2013). According to Dansi et al. (2012) these species are characterized by having been exploited under mechanisms of use such as collection, incipient cultivation, that is, characterized by a low level of production, using simple technology, focused mainly on self-consumption, and with the capacity to sustain only small populations (Zent and Zent, 2012) or being encouraged, that is, strategies applied to maintain or increase the population of useful wild or weedy plants within wild or human-made environments (Casas et al., 2007), and by their link to traditional production systems, mostly under subsistence levels; additionally, for being adapted to unfavorable heterogeneous climatic and soil conditions and, because these are more resistant to local pests and diseases (Padulosi et al., 2013).

This group of species is relevant due to its nutritional value similar to or greater than that of commercial crops (Padulosi et al., 2019; Karthiga et al., 2022), have social importance (they reaffirm cultural identity), economic importance (income opportunity for the farmer and for other actors involved in the value chain) (Padulosi et al., 2013), and productive importance (improves the resilience of the production system to biotic and abiotic factors, as these are more diverse systems) (FAO, 2018).

However, many of these species are at risk of disappearing along with the associated traditional knowledge due, among other factors, to the change from the traditional production system to one that promotes monoculture and the use of agrochemicals such as herbicides, leading to the displacement of native genotypes of the main crops and of wild or semi-domesticated species linked to traditional systems.

Furthermore, their stigmatization as a food for the poor has led to a decline in their consumption and replaced by modern products for export or import, which discourages their conservation and use in rural communities where these are found. This, combined with the focus on the production of the main species, has marginalized them in research centers, by not including them in basic crops (Padulosi et al., 2013; Nixwell et al., 2022).

This leads to a low capacity of countries to research this type of resources and to the loss of opportunity for their study, characterization and promotion as potential food resources, and as an alternative source of income mainly for poor farmers (Padulosi et al., 2013). However, in recent times, interest in the study of these species has increased, due to the aforementioned problems and the increase in demand for foods with nutraceuticals such as antioxidants.

### 2.2 Studies carried out on underutilized or abandoned species

In some regions of Southern Africa, Asia Pacific, Southern Italy and Latin America, research, production and promotion of underutilized or abandoned species has increased, increasing their popularity for food, medicinal and functional use due to their health benefits.

In species such as amaranth (*Amaranthus tricolor* L.), a significant content of antioxidant compounds such as polyphenols, flavonoids and carotenoids are reported (Platel, 2020). In buckwheat (*Fagopyrum esculentum* Moench), a high content of phenolic compounds and balanced content of amino acids and minerals, as well as high protein quality (Jing et al., 2016). In mung bean [*Vigna radiata* (L.) R. Wilczek], it is also recognized as a good source of protein, with high digestibility and suitable as baby food (HanumanthaRao et al., 2016).

In arugula (*Eruca vesicaria* L.) (Proietti et al., 2021) and *Salicornia* spp. (L.) Parl (Scarano et al., 2021), an important nutritional content and nutraceutical compounds have been recorded. Moringa (*Moringa oleifera* L.) has an important foliar content of Ca, K, Fe, Mg, P, Zn, vitamins A, B1 (thiamine), B2 (riboflavin) and B3 (niacin) (Nixwell et al., 2022). Quinoa [*Chenopodium quinoa* (Willd.)] stands out for its folic acid content, which is deficient in a large part of the world's population, and for not containing gluten (Schoenlechner, 2017), in addition to its nutritional and functional value due to its content of amino acids and antioxidants (Basantes-Morales et al., 2019).

Regarding the positive effect on health, buckwheat seeds [*Fagopyrum esculentum* (Moench)] have potential as a functional and medicinal food (Jing et al., 2016). Moringa has been documented to have an effect on reducing blood sugar and cholesterol (Nixwell et al., 2022); while *Cyclopia* (Vent.) spp. has a beneficial effect on reducing the risk of cardiovascular diseases (Windvogel, 2020). Studies carried out on tassel hyacinth [*Leopoldia comosa* (L.) Parl.] suggest that it could be an important source of compounds capable of suppressing the absorption of fat from the diet, an important alternative to drugs used to control obesity, which have been reported adverse effects (Marrelli et al., 2019). In purslane (*Portulaca oleracea* L.) an important content of omega 3 and 6 fatty acids has been reported, which regulate blood cholesterol levels, prevent water loss in the skin, and maintain proper function of the nervous system (Mera-Ovando et al., 2018).

Regarding the study to determine its response under cultivation conditions, in natal ginger [*Siphonochilus aethiopicus* (Schweinf.) B.L. Burtt], the compost extract was evaluated in the secondary metabolite content and mineral accumulation, and a positive effect on its content was reported (Jasson et al., 2023). In arugula, the effect of different light conditions on crop yield and nutritional properties was evaluated, as well as different sowing conditions and fertilization times on seedling production: it was considered that this species has potential for cultivation in controlled environment conditions (Proietti et al., 2021). In sea asparagus [*Salicornia* spp. (L.) Parl.], its importance stands out as an alternative crop in adverse environments, such as those with saline conditions (Scarano et al., 2021).

In Mexican wild husk tomato (*Physalis* spp.), different genotypes were evaluated under cultivation conditions in protected systems, with outstanding results for its production for commercial purposes (Chamú-Juárez et al., 2020). Germination studies have been carried out on species such as piquín or chiltepín chili (*Capsicum annuum* var. *glabriusculum*), since its erratic and slow germination has hindered its potential as a crop (Quintero et al., 2018). For the evaluation of new varieties of cape gooseberry (*Physalis peruviana* L.), a species recognized for its tolerance to different environmental conditions and as an alternative source of income for the farmer, Orozco-Balbuena et al. (2021) evaluated the phenology, morphology and development of four varieties under greenhouse and hydroponic conditions, with three different concentrations of nutrient solutions.

Foliar application of moringa leaf extract was evaluated as an organic biostimulant in several crops, with a favorable effect on the development of plant height, fresh and dry weight of roots, and shoots (Karthiga et al., 2022).

Honey bush [*Cyclopia* (Vent.) spp.], a species native to southern Africa, is one of the few native species that has transitioned from a wild product to a commercial one, due to its antioxidant content, an aspect of interest in the population for health-promoting foods, a factor that promoted the development of its cultivation (Joubert et al., 2011). Since 2000, the demand for quinoa and products derived from this crop has increased due to its nutritional and functional value (Schoenlechner, 2017). In huauzontle (*Chenopodium berlandieri* spp. *nuttalliae*) the starch of the seed was characterized to determine its possible use in the food industry, with acceptable results (Assad-Bustillos et al., 2014).

For the genetic improvement of buckwheat, molecular studies were carried out with molecular marker-assisted breeding, using random amplified polymorphic DNA markers (RAPD) and amplified fragment length polymorphism markers (AFLP) (Woo et al., 2016); and phylogenetic analyses were performed to define its evolutionary process, using chloroplast genome information and microsatellite markers to complement morphological descriptors (Cheng et al., 2020). In the genus *Jaltomata*, the application of DNA sequencing and phylogenetic trees allowed to define the taxonomic status of a specimen of *Jaltomata*, recognizing it as a new species for the genus (Flores-Sánchez et al., 2024). In mung beans, a genetic characterization of quantitative and qualitative traits was carried out, and molecular markers were developed (Mehandi et al., 2019). In purslane, participatory improvement programs have been established with producers, the cultivation of the species has been promoted and there are five varieties registered in Mexico (Solis, 2014).

The use of molecular techniques for domestication and development of new crops, such as marker-assisted genetic improvement, can be useful when working with underutilized wild or semi-domesticated species, because it allows studying gene expression and identifying genes of interest associated with phenotypic characteristics, performing selection (Vlk and Řepková, 2017), and reducing time and costs in the domestication process (Thulin et al., 2017), since published works on this process report that in classical breeding more than 20 generations are required to change phenotypes and obtain the expected results (Mehdi and Villamar-Torres, 2017; Fernie and Yan, 2019).

In pennycress (*Thlaspi arvense* L.) several different next-generation sequencing (NGS) strategies were used to *de novo* assemble a draft genome for the domestication of the species (Dorn et al., 2015), for celery (*Apios americana* Medik) RNA-sequencing was used to identify marker-trait associations using single-nucleotide polymorphisms (SNPs) (Belamkar et al., 2016), and in wheatgrass (*Thinopyrum intermedium* (Host) Barkworth & D.R. Dewey) genotyping-by-sequencing was used to identify markers using SNPs (Kantarski et al., 2017 *)*.

As can be seen, important work has been carried out in the study of underutilized species, where the nutritional and nutraceutical value stands out, which could contribute to solve the health problem in society; however, due to the wide diversity of species that make up this group, it is necessary to join forces to contribute to their study and to the development of a methodology that serves as a guide to direct the study and generation of information on these species, allowing the development of alternative crops, that can be defined as those different than the major crops widely produced, belonging to cereals, legumes, vegetables, root and tubers, and fruits, with greater capacity of adaptation to unfavorable environmental conditions, low-input requirements, greater tolerance to pest and diseases, high nutritional and nutraceutical value with a long-term positive effect on human health, and economic importance as source of income, important characteristics mainly for poor farmers and for consumers (Kumar et al., 2022; García-Tejero et al., 2023; Tadele, 2023).

### 2.3 Agriculture in controlled environment

Technology such as greenhouses, substrates (culture media) and hydroponics, are used in controlled environment agriculture to control the factors that influence the development of a crop in its characterization and evaluation process (Bethke and Lieth, 2016), to determine the viability of new plant genetic resources for agricultural purposes, because it facilitates the study of the plant behavior in its growth and development in the face of changing environmental factors (Both et al., 2015; Bustamante et al., 2016).

Factors such as light, temperature, relative humidity, CO_2_ level, presence of pests and diseases, are possible to control with the use of greenhouses (Singh et al., 2015; Leite et al., 2017). Of these, light and temperature are probably the factors that most affect plant growth and development (Franklin et al., 2014).

The response to temperature depends on the species, with an optimal interval in the different stages of plant development (Hatfield and Prueger, 2015), outside of which, it presents different and diverse reactions to withstand the stress generated by this factor (Yang et al., 2018), such as the expression of protective proteins such as heat shock protein 70 (HSP70) (Dickinson et al., 2018), where a calcium-conducting cyclic nucleotide gated channel (CNGC2) has a function to induce HSPs proteins (Franklin et al., 2014), response that is coordinated by heat shock factors (HSFs) (Dickinson et al., 2018).

Light, used as an environmental signal and in the process of photosynthesis (Jones, 2018), is perceived by different types of photoreceptors linked to signaling pathways such as phytochromes, cryptochromes, phototropins and UVR8. In combination with a chromophore, these photoreceptors define the light absorption properties (Thoma et al., 2020).

With inert substrates, the concentration of nutrients in the root zone can be controlled (Silber and Bar-Tal, 2008), because these do not contribute with nutrients, absorption or ionic exchange (Raviv and Lieth, 2008), unlike soil, where microenvironments are generated that influence nutrient bioavailability (Nguyen et al., 2016).

Hydroponics uses nutrient solution (NS) to grow plants with or without the use of substrate, where the nutrients are supplied (Nguyen et al., 2016; Sharma et al., 2018). Factors such as pH and electrical conductivity (EC) are important in NS, and pH influences the availability of nutrients to the plant (Sánchez et al., 2022), with an optimum pH of 5.5 to 6.5 for most species (Alexopoulos et al., 2021). EC is crop-specific and is related to the amount of ions available to plants (Ding et al., 2018), an important aspect, because a nutrient excess or deficiency affects the development and yield of the plant (Lam et al., 2020).

### 2.4 Methodological and research needs

According to Sarukhán et al. (2017), an important requirement for the management, use and conservation of biodiversity is a deep knowledge of the species.

FAO, in the Second Global Plan of Action for Plant Genetic Resources for Food and Agriculture (FAO, 2012), mentions the importance of generating data on the characterization and evaluation of plant genetic resources, and identifying germplasm with possibilities of improvement or direct use for production and commercialization. Furthermore, it emphasizes the obstacle represented by the lack of adequate data in these aspects to use these resources, particularly underutilized species.

Additionally, FAO (2012) refers to the areas in which research programs should intervene, such as genetic characterization to identify useful genes, understanding their expression and variation, improvement to increase yield in quantity and quality, nutritional quality, integration of abandoned or underutilized species in diversified production systems, development of value chains and awareness about nutritional value (FAO, 2018).

However, one of the difficulties for the study, evaluation, production and promotion for consumption of underutilized or abandoned species is mainly the development of skills in people interested in these resources, to address the challenges of interdisciplinary research and integrate the nutritional aspect in agricultural development, a difficulty that is increased by the lack of a standard methodology for the study, evaluation and selection of germplasm for improvement (Padulosi et al., 2013).

Therefore, the following topics are proposed, which are considered basic to begin the study of underutilized plant genetic resources and develop alternative crops, with the aim of contributing to overcoming the problem raised, through the evaluation of these resources from agronomy perspective in connection with genetics and botany.

The proposed theme can serve as a methodological guide and be followed sequentially, or focus on those aspects that are considered necessary, according to the level of study on the target species.

## 3 Basic topics in the evaluation of plant genetic resources to generate alternative crops

These topics can be applied according to the level of study of the species of interest to resolve the missing aspects. In species that have not been studied for agricultural production purposes, it is recommended to prioritize in order to make efficient use of resources, based on the criteria to consider new plant genotypes for food purposes: edible plants for humans, with high nutritional value, that do not require intermediate processes between cultivation, harvest and use (Flores-Sánchez et al., 2021) climate adaptation, cultural importance, risk of genetic erosion, alternative income generation, importance in the ecosystem (Ulian et al., 2020), content of nutraceutical compounds or secondary metabolites and level of domestication.

The selection of criteria will depend on the interests of the researcher, but it is recommended to prioritize nutritional aspects and nutraceutical compound content.

The proposed theme include aspects of seed germination, traits of agronomic interest, nutrient absorption, phenology, fruit quality and secondary metabolite profile.

### 3.1 Seed germination evaluation

The seed is the main way of dispersion of plants in the environment (Arc et al., 2011) and is the main way in most crops to start a new production cycle (Finch-Savage and Bassel, 2015). The ability for rapid germination and development, early and uniform plant emergence, is desirable for productive purposes, since this capacity influences yield, quality and economic income (Quintero et al., 2018; Mirmazloum et al., 2020).

Obtaining these germination characteristics is one of the problems when working with wild or semi-domesticated species, because the dormancy mechanisms presented by the seeds of some of these species, a characteristic that prevents germination and allows them to persist under unfavorable conditions (Flores-Sánchez et al., 2022).

In general, two types of dormancy are recognized: endogenous, where some characteristics of the embryo limit germination; and exogenous, where structural and chemical characteristics of the seed prevent this process, so, considering physical, morphological, physiological aspects, and the combination of these, dormancy can be classified into five classes, with non-deep physiological dormancy being the one that occurs in most seeds (Baskin and Baskin, 2014). To overcome this problem, techniques such as physical and chemical scarification, stratification, and the combination of these are used to help the seeds germinate, using different materials and equipment depending on the type of dormancy (Flores-Sánchez et al., 2022).

With physical and chemical scarification, the aim is to promote or accelerate germination by facilitating the entry of water, by increasing the permeability of the seed coat or testa. In the first, abrasion, cutting, partial removal of the testa or immersion in hot water are used, and in the second, the use of chemical substances such as sulfuric (H_2_SO_4_) or nitric (HNO_3_) acids.

In barrel cactus [*Echinocactus parryi* (Engelm.)] it was reported that removing 50% of the testa resulted in the best germination percentages, compared to gibberellic acid (GA_3_), a plant development regulator, and sulfuric acid (García-González et al., 2022). In porknut (*Vachellia macracantha* (Humb. & Bonpl. ex Willd.) Seigler & Ebinger), the abrasion of the testa gave better results than chemical scarification (Maldonado-Arciniegas et al., 2017). On the contrary, in *Conanthera* spp., with physical scarification of the seed by abrasion, no effect on seed germination was reported (De la Cuadra et al., 2019).

In stratification, cold and warm temperatures are used, individually or in combination. In mountain crowberry (*Empetrum hermaphroditum*), warm stratification treatment followed by cold stratification is required to obtain the highest percentage of germination (Baskin et al., 2002); while, in japanese rose seeds (*Rosa rugosa*), the germination process is favored with cold stratification followed by warm stratification (Gao et al., 2022).

In addition to these treatments, development regulating substances such as GA_3_ are used, a natural plant regulator with different applications in agriculture, such as seed germination (Cornea-Cipcigan et al., 2020), in addition, ascorbic and salicylic acids are used, with reported effects on germination (Dotto and Silva, 2017).

Also, development-promoting chemical substances such as potassium nitrate (KNO_3_), phosphate (KH_2_PO_4_) or chloride (KCl), or polyethylene glycol (PEG) are used through the seed imbibition in a solution with a certain concentration, controlling the conditions between the water potential of the solution and the water potential within the seed during the hydration process, which favors the rapid emergence capacity of the radicle (Moaaz et al., 2020).

The results obtained will depend on the species and the technique used. In caraway (*Carum carvi* L.), it was reported that PEG had the best effect on germination, followed by KNO_3_ and KCl (Mirmazloum et al., 2020). In seeds of tomato cultivars (*Solanum lycopersicum*), Sundar and Ahmar, it was recorded that a concentration of 0.75% of KNO_3_ promoted germination (Moaaz et al., 2020). In beet (*Beta vulgaris*), it was determined that treatment with water was the most effective, followed by salicylic or gibberellic acids (GA_3_), and ascorbic acid (Dotto and Silva, 2017). For the germination of habanero chili seeds (*Capsicum chinense* Jacq.), it was reported that abscisic acid increased the germination speed; while PEG and KNO_3_ favored the emergence of the seedling (Garruña-Hernández et al., 2014).

Another factor in germination is light. The seed has environmental sensors that allow it to identify the right time to start the germination process, such as phytochrome, cryptochrome, phototropin and zeitlupe (ZTL) photoreceptors, with phytochromes being the main ones (Yan and Chen, 2020; Footitt et al., 2013). Phytochrome-A (PHYA) mediates the response to very low fluence light (VLFR) and is present in seeds with extreme light sensitivity, whereas, phytochrome-B (PHYB) mediates the response to low fluence light (LFR) (Arana et al., 2007).

### 3.2 Traits of agronomic interest

The response of a plant species to environmental conditions different from those of its natural habitat, such as growing conditions, will be determined by the genotype and its interaction with the environment (Gerrano et al., 2019). This response can be evaluated through different traits of agronomic interest such as stem diameter, plant height, number of leaves, clusters and flowers, fruits per cluster and fruit weight (Flores-Sánchez et al., 2021).

The evaluation of these traits makes it possible to assess the viability of a species and the appropriate conditions for its production in a cultivation environment.

#### 3.2.1 Plant height

Plant height is important as it is related to architecture, lodging resistance (resistance of the aerial parts of the plant to the horizontal-permanent condition from its vertical position due to factors such as wind or rain) and yield (Juárez-López et al., 2012; Gao et al., 2020). In tall species or indeterminate growth height is relevant under greenhouse conditions, because it influences the risk of damaging stems and the use of labor during tying (Juárez-López et al., 2012). It can be influenced by management (elimination of axillary and basal shoots) and by EC (the higher the EC, the lower the plant height), as reported in jaltomate (*Jaltomata procumbens*) (Flores-Sánchez et al., 2021) and tomato (Marchese et al., 2008). In addition, by removing axillary shoots and leaves, or managing planting densities as recorded in tomato (Casuriaga et al., 2020; Jo and Shin, 2020).

#### 3.2.2 Stem diameter

Stem diameter is a characteristic related to the plant's ability to withstand the stress generated in the transplant, lodging resistance, and is also an indicator of plant vigor (Antúnez-Ocampo et al., 2014; Chiquito-Contreras et al., 2018), which expresses the accumulation of reserves that can be transferred to sites of demand (Preciado-Rangel et al., 2002), where leaf area and metabolic activity are important for the synthesis and accumulation of these reserves in the stem, during vegetative development (Pegoraro et al., 2014).

This characteristic can be influenced by plant management, as reported in jaltomate (Flores-Sánchez et al., 2021), where pruning of lateral and basal shoots reduced stem diameter, while high EC of NS increased it, effect that was attributed to the availability of N in the culture medium, an element involved in the development of leaf area and therefore, in the synthesis of photosynthates (Leghari et al., 2016). Furthermore, it is attributed to the presence of K, an element involved in stomatal opening, cell expansion, enzymatic activation and osmotic adjustment, whose absorption is often closely correlated with that of N (Ragel et al., 2019). Stem diameter can also be affected by the application of growth regulators such as GA_3_, which in bean (*Phaseolus vulgaris* L.) reduced stem diameter (Troyjack et al., 2017).

#### 3.2.3 Number of leaves and leaf area

The number of leaves is one of the factors on which the photosynthesis and yield of the plant depend (Jo and Shin, 2020). It is also an indicator of the leaf area where the carbon skeletons that the plant uses in its different structures or that are stored in the stem are produced (Preciado-Rangel et al., 2002).

This characteristic can be influenced by the EC of the NS. High EC levels cause water stress in the plant, due to the decrease in the osmotic potential of the NS by increasing the concentration of nutrients in the root zone, which can affect the absorption of water and some nutrients (Bagale, 2018; Parra et al., 2008), and therefore, the vegetative development of the plant as reported in the number of leaves in jaltomate (Flores-Sánchez et al., 2021) and tomato (Bustomi et al., 2014).

#### 3.2.4 Reproductive structures characteristics

Characteristics such as the number of clusters, flowers and fruits, as well as the fruit weight are related to the yield of the plant, an important aspect in crop production. In species such as tomato and cowpea (*Vigna unguiculata* L.), it has been reported that yield is determined by the number and weight of fruits, and by the number of pods related to the number of flowers, respectively (Sánchez-Del-Castillo et al., 2014; Gerrano et al., 2019). These structures can be affected by plant management, through pruning or by the EC level of the NS.

As for pruning, the elimination of shoots allows regulating vegetative and reproductive growth, favoring the generation of flowers and fruits, and the removal of reserve substances towards the fruits, the main organs of demand in the flowering and fruiting stage, promoting higher fruit weight (Ponce-Valerio et al., 2011; Mbonihankuye et al., 2013). High EC negatively influences the number of clusters, flowers and fruits (Marchese et al., 2008), and the fruit weight according to the genotype (Colli-Cortés et al., 2020), as fruit expansion is affected by a decrease in osmotic potential (Dorai et al., 2001).

### 3.3 Phenological characteristics

Phenology studies the stages of the life cycle of organisms and how they are synchronized as the time and climate change (Liang, 2019). They occur naturally during plant growth, and their study seeks to evaluate the variations in the moment in which these stages occur. By identifying correlations between environmental changes and particular developmental events, the plant's reaction to the environment is described and, in addition, an attempt is made to predict its reaction under new environmental conditions (Keller, 2020).

Knowledge of the phenological stages and their variability is useful in crop management, in the selection of materials for a given area (Chmielewski, 2013), and in the study of new plant genetic resources for use in agricultural purposes, because each genotype will respond differently to different environments (Córdoba et al., 2018), and to the production systems and technology used, influencing its adaptation and production (Sabino-López et al., 2016).

Studies on these characteristics have been carried out on species such as cocoa (*Theobroma cacao* L.) (Meneses-Buitrago et al., 2019), tomato (Córdoba et al., 2018), and cape gooseberry (Sabino-López et al., 2016), in which the differential response in the emission of floral structures and fruit formation has been documented, in greenhouse and field conditions.

### 3.4 Nutrient uptake

In any production system, the balanced supply and availability of nutrients are the most important factors, because nutrient excess or deficiency will affect the physiology, potential growth and yield of the crop (Lee et al., 2017; Mardanluo et al., 2018).

In hydroponic systems, the nutrients required by the plant are supplied through the NS (Nguyen et al., 2016), with a specific concentration of nutrients measured by the EC. The EC is proportional to the total ions contained in said solution, where availability and balanced supply are fundamental aspects to improve crop production (Lee et al., 2017; Ding et al., 2018).

With the use of NS, it is possible to control the concentration of nutrients, evaluate their absorption by the plant (Raviv and Lieth, 2008), and generate absorption curves. Genetic factors, phenological stage and environmental conditions (González et al., 2018) influence the processes of absorption, transport, and nutrient storage based on the requirements of each plant structure (Gilliham et al., 2011; Guo et al., 2016; González-Fontes et al., 2017); therefore, they are essential for the design of fertilization plans (Castro-Villarreal and Villarreal-Núñez, 2020).

The information obtained, which is genotype-dependent and differentially expressed according to the evaluated structure is relevant when new plant genetic resources are studied for use in agricultural production systems (Gandica and Peña, 2015). In black pepper (*Piper nigrum*), the leaf and cluster evaluation showed a variation in the first structure during the growing season, mainly in the accumulation of K and Mg; while, in the second, the greatest accumulation was recorded for N, K and Ca (Dalazen et al., 2020). In cape gooseberry, the elements reported with the highest accumulation in leaves were N, P, Ca and Mg; in stems it was K (Torres et al., 2015).

### 3.5 Fruit quality

Attributes such as firmness, total soluble solids (TSS) concentration, color, titratable acidity (TA), size, fruit weight and the ratio of TSS/TA are some important characteristics to determine fruit quality, and influence its acceptance by the consumer.

The attributes considered to define quality will depend on the fruit type and destination market. Thus, for example, in blueberry (*Vaccinium corymbosum*) the industry considers color, aroma, sweetness, acidity and firmness to be important (Bolaños-Alcántara et al., 2019) and the wax that covers the fruit (bloom), whose function is to protect it from desiccation or damage by pathogens (Loypimai et al., 2017). On the contrary, uniform fruit color, sweetness, flavor, juiciness and antioxidant content are important to consumers (Gilbert et al., 2014). In tomato, the industry considers dry fruit weight, pH, TA, TSS, viscosity and color to be important; while the consumer focuses on the color, size, flavor and firmness (Islam et al., 2019; Luna-Flete et al., 2018).

These attributes are determined by the combination of nutritional, environmental (light and temperature), physiological, genotype, water stress or type of plant management factors (Wiesler, 2012).

The nutritional factor can influence characteristics such as fruit firmness, TA and TSS. Elements such as B, Ca, Se, and Si, are related to firmness (Rahman et al., 2021) by influencing the properties of the cell wall, and has been documented in tomato (Preciado-Rangel et al., 2021) and apple tree (*Malus* × *domestica* Borkh) (Karagiannis et al., 2021). On the other hand, it is reported that K has an effect on TA because it reacts with organic acids and decreases their content (Zhang et al., 2018). Also, it was reported that the interaction of N and S (elements that influence post-harvest quality) positively affected the TSS content in tomato (Siueia et al., 2020).

Light conditions affect fruit size and TSS by affecting the photosynthetic apparatus and influencing the supply of photoassimilates to the fruit, increasing or reducing its size (Gruda, 2005) and TSS content. In strawberry (*Fragaria* × *ananasa* Duchesne), in a pyramidal cultivation system, it was reported that the TSS concentration decreased as the shade on the plant increased (Alvarado-Chávez et al., 2020).

The water stress generated by salinity decreases the fruit size, caused by a reduction in water potential because less water flow to fruit, decreasing its expansion rate (Salas-Pérez et al., 2016); opposite effects indicate tolerance to salinity (Flores-González et al., 2012).

Genetic aspects determine characteristics such as fruit size. Larger fruits have been reported in plants with fewer of them, which influences a greater distribution of dry matter related to stored photoassimilates and which maintain favorable water potentials as the cell expands (Pérez-Labrada et al., 2016; Thomas, 2017). They also influence the plant's ability to tolerate salinity, allowing it to express its potential regardless of the level of stress to which it is subjected (Flores-González et al., 2012).

Plant management affects aspects such as fruit size and quality. Management practices such as pruning allow regulating vegetative and reproductive growth by promoting the removal of reserve substances towards the fruits, which helps to improve their quality with greater size and TSS content, and lower TA (Casuriaga et al., 2020; Falchi et al., 2020).

The condition of high TSS values and lower TA is important to obtain a high TSS/TA ratio, reflected in greater palatability of the fruit that influences the product to be more accepted by consumers (Batista-Silva et al., 2018; Santacruz-Oviedo et al., 2018).

### 3.6 Secondary metabolites

Secondary metabolites, phytochemicals that are not directly linked to normal plant development, perform defense functions against pathogens and herbivores, and protection against abiotic stress factors such as low temperatures, drought and UV-B radiation (Moore et al., 2014; Anulika et al., 2016).

Based on their chemical structure and biosynthetic pathway, they can be classified into terpenoids, phenolic and nitrogenous compounds (Yeshi et al., 2022). In humans, functions such as antioxidant, anti-inflammatory, antifungal, anticancer, antimicrobial, neuroprotective, and in the treatment of diabetes, high blood pressure, and kidney stones are described (Panche et al., 2016; Kumar and Goel, 2019; Ashraf and Bhatti, 2021).

The content of secondary metabolites will depend on the genotype, the climate in which the plant grows, and the agricultural practices applied (Liu et al., 2021). Factors such as temperature, light, growing conditions such as salinity and pruning management, generate stress in the plant and promote enzymatic activity and overexpression of genes in the antioxidant system, influencing the synthesis of metabolites (Martinez et al., 2016; Ding et al., 2018).

Its study and characterization are important to evaluate the plant response to environmental changes, biotic and abiotic stress factors (Ibarra-Estrada et al., 2016; Sousa et al., 2019), which can help in the evaluation of new plant genetic resources, wild or semi-domesticated, for their production under growing conditions and to determine the positive or negative effects on the contents of these metabolites.

In this way, the proposed theme seeks to serve as a methodological guide for the evaluation and characterization of underutilized or abandoned plant genetic resources, which allows generating the necessary data to follow up on the study of said resources for their improvement or, for their direct use through production and marketing.

It also seeks to meet the needs raised by the WHO and those mentioned in the Second Global Action Plan for Plant Genetic Resources for Food and Agriculture, and in the second objective of the Sustainable Development Goals.

## 4 Methodology

Next, the methodology that covers the points mentioned is developed in a general way, and the one implemented by Flores-Sánchez (2022) in the evaluation of jaltomate is taken as an example.

### 4.1 Germination

Under natural conditions, the seed has environmental sensors that allow it to identify the appropriate germination time, therefore, for agricultural use the combination of light, temperature and the use of growth-promoting substances is recommended, with different levels for each factor. Also, according to the characteristics of the seed coat, the aforementioned techniques can be used for their evaluation.

KNO_3_ as an example, is used in concentrations in the range of 0.1 – 1%, although higher concentrations can be used considering the species to be treated, as reported by Moaaz et al. (2020) and Mutetwa et al. (2023) in tomato and kiwano (*Cucumis metuliferus*), respectively. For the use of substances such as KCl and PEG_6000_, the methodology described by Mirmazloum et al. (2020) can be consulted, and for the use of GA_3_, the methodology reported by Cornea-Cipcigan et al. (2020). Seed imbibition times can vary from 1 h to 6 days, depending on the species (**Table 1**).

**Table 1:** Germination-promoting substances and imbibition times used in different plant species.

Plant species	Germination-promoting substances				Imbibition time (h)
	KNO_3_	KCl	PEG_6000_	GA_3_	
*Cannabis sativa* L. (Langa et al., 2024)	✓			✓	1 – 24
*Cucumis metuliferus* E. Mey. Ex Naudin (Mutetwa et al., 2023)	✓				12
*Jaltomata procumbens* (Cav.) J.L. Gentry (Flores-Sánchez et al., 2022)	✓				96 – 144
*Cannabis sativa* L. (Du et al., 2022)				✓	8
*Solanum lycopersicum* L. (Moaaz et al., 2020)	✓				24
*Carum carvi* L. (Mirmazloum et al., 2020)	✓	✓	✓		12 – 36
*Sorghum bicolor* (L.) Moench (Patane et al., 2016)			✓		48
*Capsicum annuum* var. *glabriusculum* (Quintero et al., 2018)	✓	✓		✓	24 – 72

Once the imbibition period is over the seed is removed from the substance, placed and distributed in containers such as Petri dishes, with filter paper previously moistened with distilled water. The seed is kept during the evaluation period in a controlled environment chamber, which allows maintaining the levels of the factors established for the treatments, such as light and temperature, applying distilled water to keep the seed moist.

The seed is monitored daily, and germination is recorded considering this at the time of radicle emergence, greater than 2 mm in length. Germination variables are calculated with the data obtained:

Germination percentage (GP):


$$\text{GP}=\;\frac{Number\;of\;germinated\;seeds}{Number\;of\;seeds\;set\;to\;germinate}\times 100$$

Energy period (EP): days after treatment to reach 50 % or more of germinated seed.

Germination energy (GE): cumulative germination percentage once reached the energy period.


$$\text{GE}=\frac{Cumulative\;daily\;total\;of\;germinated\;seeds}{Number\;of\;seeds\;set\;to\;germinate}\times 100$$

Germination rate (GR): number of germinated seeds every day.


$$\text{GR}\begin{array}{c} =\frac{Number\;of\;germinated\;seeds}{Number\;of\;days\;to\;first\;count}\\ +\ldots +\frac{Number\;of\;germinated\;seeds}{Number\;of\;days\;to\;last\;count} \end{array}$$

For statistical analysis of the data, it is recommended to apply logistic regression analysis for the germination percentage and analysis of variance with means separation test for the germination variables, applying data transformation in case they do not meet the statistical assumptions.

### 4.2 Traits of agronomic interest

The data collected in this stage are used to evaluate characteristics considered of agronomic interest such as plant height, stem diameter, number of leaves and leaf area, as well as reproductive structures; however, to make efficient use of resources, data on phenological characteristics, fruit quality and secondary metabolite content can also be obtained for evaluation.

The plant species to be characterized and evaluated must be established under greenhouse and hydroponic conditions. This makes it possible to control environmental factors that could affect the development of this activity. In addition, the use of inert inorganic substrates is important, since they allow controlling the concentration of nutrients in the root zone by not contributing with nutrients, absorption or ion exchange.

If a substrate needs to be evaluated for use, a physical and chemical evaluation can be carried out to select the most suitable one. In general, the minimum physical and chemical properties recommended for the selection of a substrate are shown in **Table 2**.

**Table 2:** Minimum physical and chemical properties for the selection of inorganic substrates for plant growth.

Property	Optimal level
1. Particle diameter (mm)	0.25 - 2.5
2. Total porosity (%)	> 85
3. Water readily available (%)	20 - 30
4. Aeration capacity (%)	20 - 30
5. Bulk density (g cm^-3^)	0.05 - 0.8
6. Cation exchange capacity (CEC) (cmol_(+)_ kg^-1^)	< 10
7. Electrical conductivity (EC) (dS m^-1^)	< 0.15

Table elaborated based on Abad et al. (1993); Abad et al. (2005) and Castellanos and Tapia (2009).

The container to be used will depend on the species to be evaluated. Among the types of containers that can be used are black polyethylene bags 40 × 40 cm (13 L) or 35 × 35 cm (9 L).

For plant nutrition, to determine the most suitable level for the species under evaluation, it is recommended to use the Steiner nutrient solution (**Table 3**, Steiner, 1984) with different nutritional levels, measured through the EC: low, sufficient and high. Although there are other nutrient solutions that can be consulted in Trejo-Téllez and Gómez-Merino (2012).

**Table 3:** Macronutrients and micronutrients of the nutrient solution according to Steiner (1984) for an electrical conductivity of 2 dS m^-1^ (osmotic potential -0.072 MPa).

Macronutrient [mol_(+)_ m^-3^]					
NO_3_^-^	P-H_2_PO_4_^-^	K^+^	Ca^2+^	Mg^2+^	S-SO_4_^2-^
12	1	7	9	4	7
Micronutrient (mg L^-1^)					
B	Cu	Fe	Mn	Mo	Zn
0.20 - 0.60	0.01 - 0.06	0.50 – 20.00	0.20 - 2.00	0.04 - 0.06	0.10 - 0.60

An EC level of 2 dS m^-1^ can be considered sufficient, equivalent to -0.072 MPa of osmotic potential, at which many plants are known to thrive (Steiner, 1961). This level corresponds to the application of 100% Steiner nutrient solution. From this value low and high levels can be established.

To determine the phenological behavior of the species under growing conditions, data on the start of flowering, fruiting and harvest are recorded, considering the days elapsed in each stage after transplanting.

In addition, it must be evaluated whether the species presents self-fertilization characteristics and if it requires any practice for pollination to occur. If this characteristic is previously known, it is evaluated whether the growing conditions affect it positively or negatively.

Some agronomic characteristics can be evaluated, such as plant height (recorded with a tape measure from the base of the main stem to the tip), stem diameter (measured with a vernier graduated in mm at the height of the cotyledonary leaves), total number of leaves, type and number of inflorescences, and the total number of flowers, fruits per cluster, fruit weight and yield per plant.

When the usable part of the plant is not the fruit, measurements are adapted. For example, in the case of leaves, the most appropriate time for harvesting can be determined and the weight of the biomass generated recorded to obtain the yield.

The chlorophyll content of plant leaves is another important characteristic because it is linked to the condition of the plant, and can be estimated using a SPAD measuring device that allows estimating the relative chlorophyll content in leaves by measuring leaf greenness (Jiang et al., 2017).

At this stage, the type of plant growth is evaluated to determine the pruning and tutoring needs. If the type of growth is previously known, pruning levels can be evaluated to determine its response to this cultural work, as reported in jaltomate (Flores-Sánchez et al., 2021).

### 4.3 Fruit quality

To evaluate fruit quality, the following variables can be determined: equatorial and polar diameters of the fruit, fruit weight, TSS or degrees Brix, TA, TSS/TA ratio, pH, firmness and fruit color.

To determine equatorial and polar diameters, that is, the length and width of the fruit, a vernier caliper can be used in centimeters. The fruit weight can be determined with a precision digital scale in grams. Firmness is measured with a fruit penetrometer at two equatorial parts of the fruit and the data are reported in Newtons (N).

For TSS, two drops of fruit juice are placed in a digital refractometer and the data are recorded as TSS percentage. As for TA, the following methodology is applied (Association of Official Analytical Chemists (AOAC), 1995), 10 g of fruit are ground with 50 mL of distilled water, the total volume is obtained and a 5 mL aliquot is taken, three drops of phenolphthalein are added as an indicator and titrated with 0.1 N NaOH; with the data obtained, the TA value is obtained with the formula:


$$\begin{array}{c} Titratable\;acidity\;(\%)\\ =\frac{mL\;NaOH\times N\;NaOH\times meq\times VT\times 100}{A\times g} \end{array}$$

Where: mL NaOH = milliliters of sodium hydroxide spent in the titration; N NaOH = sodium hydroxide normality; meq = milliequivalents of the predominant acid in the fruit; VT = prepared sample volume; A = aliquot taken for the measurement; g = weighed grams of the fruit.

To determine the TSS/TA ratio, divide the TSS percentage by the TA percentage (Medeiros et al., 2017).

To determine the color, the methodology described by Konica Minolta (2007) can be applied. With a colorimeter, the L*, a* and b* values are recorded in two areas of the equatorial part of the fruit. The Hue angle (h) and color purity (C) are calculated using the a* and b* values ​​with the following formulas:


$${}^{\circ}\text{h}={\text{tan}}^{-1}(\text{b}/\text{a})\text{ C}=\surd (a^{2}+b^{2})$$

The lightness (brightness) value L is obtained directly from the colorimeter. With the values obtained from L*, a* and b*, the color of the fruit is located in the color space L*a*b*, described in Konica Minolta (2007).

### 4.4 Secondary metabolites

In addition to its importance in assessing plant response to environmental changes, this characteristic is relevant due to the growing demand for products with health-beneficial components.

It is recommended to carry out a metabolomic profile using high-performance liquid chromatography (HPLC) to determine the types of metabolites present in the plant, as described by Flores-Sánchez et al. (2023) to determine flavonoids, terpenoids and phenolic acids: 0.5 g of oven-dried or lyophilized material are weighed and placed in 15 mL conical centrifuge tubes, afterwards 5 mL of 80% methanol are added and shaken with a vortex for 10 s, then subjected to an ultrasound process for 30 min, at 10-min intervals, with a 5-min rest between intervals. They are centrifuge at 5000 rpm for 5 min, and 1 mL of the extraction is taken and placed in a 1 mL capacity vial. Samples are stored at 4°C until analysis on HPLC.

To identify phenolic acids and flavonoids, the wavelengths λ1 = 254, λ2 = 280, λ3 = 330 and λ4 = 365 nm are used. The absorption spectra of the analyzed samples are obtained, they are compared with those of the standards, and calibration curves are generated at the different wavelengths.

For the identification of terpenoids, the wavelength of 220 nm is used, and calibration curves are generated as described in the previous paragraph. Reference standards are shown in **Table 4**.

**Table 4:** Reference standards of phenolic acids, flavonoids, and terpenoids.

Compound				Wavelength (nm)
Flavonoids		Phenolic acids		
Rutin		Protocatechuic ac.	3,5-di-hydroxybenzoic ac.	254
Morin		p-hydroxybenzoic ac.	Vanillic or caffeic ac.	
Quercetin		b-resorcylic ac.		
Catechin	Naringenin	Gallic acid		280
Hesperidin	Phloretin	Syringic acid		
Phloridzin		p-coumaric acid		
Apigenin		Chlorogenic acid	Ferulic acid	330
		Sinapic acid	Rosmarinic acid	
Myricetin				365
Kaempferol				
Isorhamnetin				
Terpenoids				
Carnosol		Stigmasterol		
Ursolic acid		Alpha-amyrin		220
Oleanolic acid		β -sitosterol		

It is recommended to analyze the data obtained based on a factorial model, considering the different factors and levels used, especially if these represent a factor such as EC, pruning or temperature, since the synthesis of metabolites is affected by these and by the levels of each factor to which the plant is subjected.

### 4.5 Nutritional concentration

At this stage, the crop is established again with the most suitable EC determined for the species, and the macro and micronutrients contained in the leaves are quantified for each stage of plant development (vegetative, flowering, fruiting and harvesting); in addition, characteristics such as stem diameter, stem and root length, leaf area and total dry matter are evaluated.

For the quantification of macro and micronutrients, the leaves are dried (70°C, in a forced air oven) and ground in a Wiley-type mill (particles < 2 mm). Then 0.25 g are weighed and subjected to wet digestion at 300°C, with 2 mL of a 2:1 (v/v) mixture of sulfuric acid (H_2_SO_4_) and perchloric acid (HClO_4_), and 1 mL of hydrogen peroxide H_2_O_2_ at 30%. Each sample is then diluted to obtain 25 mL with deionized water and filtered.

The N is determined with the semimicro-Kjeldahl method (Horneck and Miller, 1997), by which an aliquot of 10 mL of the sample obtained is distilled and titrated with 0.05 N sulfuric acid (H_2_SO_4_, 0.05 N). For titration, 20 mL of boric acid at 4% and 6 drops of the indicator solution are added [bromocresol green (0.3 g) and methyl red (0.165 g) in 400 mL of 95% ethanol, and the solution is taken to final volume of 500 mL].

To calculate the percentage of total N, the following formula is applied:


$$\%\;\text{N}=\frac{\text{V}\;*\;\text{N}\;*\;14\;*\;100}{\text{Sample weight }(\text{g})}$$

Where: % N = percentage of total N, V ​​= volume spent in the titration, N = normality of H_2_SO_4_, 14: milliequivalent weight of N (mg).

For the other macronutrients (P, K, Ca and Mg) and for the micronutrients (B, Cu, Fe, Mn, Mo and Zn) a coupled plasma induction atomic emission spectrophotometer is used for their quantification (ICP-AES 725-ES, Agilent, Santa Clara, CA, USA).

For the determination of sulfur (S), a wet digestion is carried out at 160°C, using 0.5 g of material with 5 mL of a 2:1 (v/v) mixture of nitric acid (HNO_3_) and perchloric acid (HClO_4_). Afterwards, each sample is diluted to 25 mL with deionized water and filtered. In this sample, except for N, the nutrients including S are determined in the coupled plasma induction atomic emission spectrophotometer.

**Figure 1:**
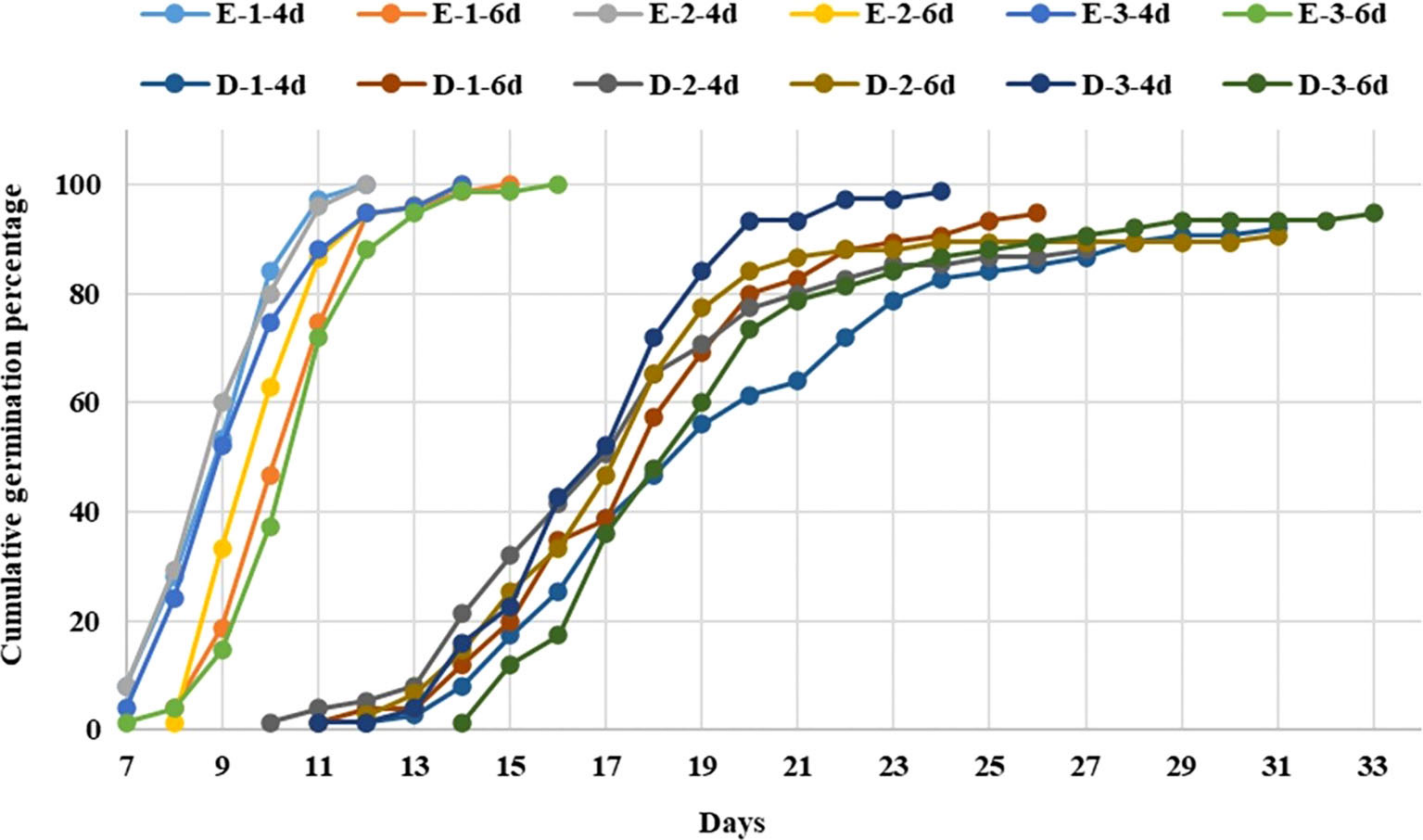
Logistic regression analysis applied to the germination percentage in two genotypes of jaltomate using the Wald test. Population: erect E, erect; D, decumbent; solution: 1 (0.1 % KNO_3_), 2 (0.2 % KNO_3_), 3 (control); imbibition: 4 days (4d), 6 days (6d) (modified from Flores-Sánchez et al., 2022).

**Figure 2:**
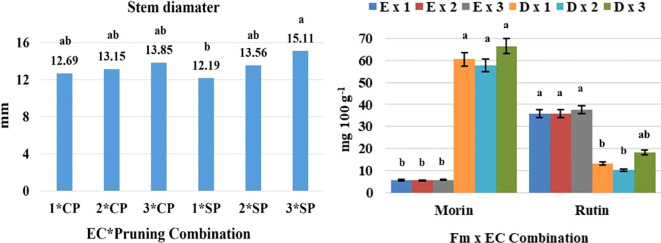
Analysis of variance based on a completely randomized factorial model and means separation test, applied to variables of agronomic interest such as stem diameter (left) and secondary metabolites (right): WP, with pruning; WoP, without pruning; Fm, Form; EC, electrical conductivity; population: E, erect; D, decumbent; different letters among bars indicate statistical difference; the thin black bars in the image on the left indicate the standard deviation, and in the image on the right indicate the standard error (modified from Flores-Sánchez et al., 2021 and Flores-Sánchez et al., 2023).

**Figure 3:**
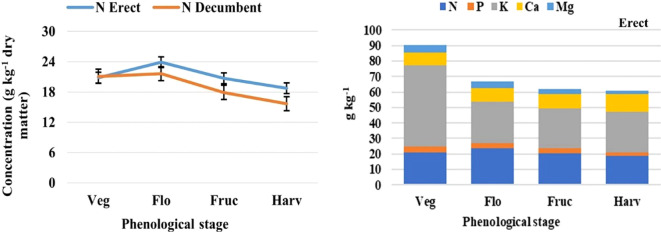
Analysis of variance based on a completely randomized factorial model, applied in nutrimental concentration, to determine the nitrogen (N) concentration by vegetative stage (left), and the order of the level of macronutrients uptake (right). Phenological stage: Veg, vegetative; Flo, flowering; Fruc, fruiting; Harv, harvest; the thin black bars in the image on the left indicate the standard error (modified from Flores-Sánchez, 2022).

**Figure 4:**
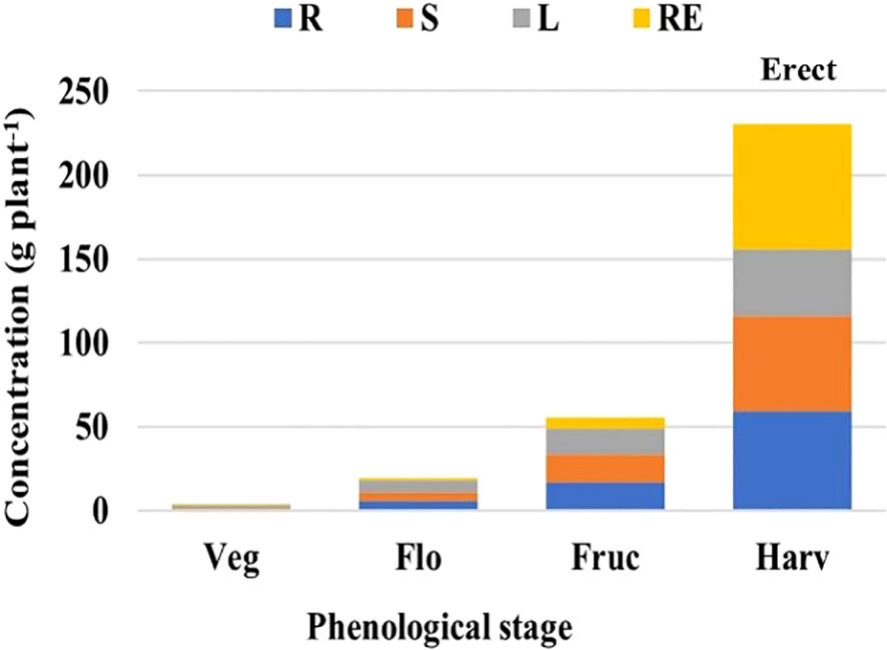
Analysis of variance based on a completely randomized factorial model, applied in nutrimental concentration, to determine the accumulation of dry matter by evaluated plant organ and by vegetative stage. Erect population; R, root; S, stem; L, leaf; RE, reproductive structure; phenological stage: Veg, vegetative; Flo, flowering; Fruc, fruiting; Harv, harvest (modified from Flores-Sánchez, 2022).

Once the evaluation and characterization of underutilized plant genetic resources is completed with this proposal, the data obtained can be analyzed by logistic regression for the case of germination percentage and, for the germination variables, agronomic assessment and nutrient concentration, analysis of variance with means separation test can be used. In addition, if different factors and levels are used for each factor, it is recommended to carry out the analysis based on a factorial model. As an example, the data presentation can be done as shown in the following figures (**Figures 1**–**4**).

## 5 Benefits of the proposal

The proposal raises different criteria that could serve as a guide for selecting plant species and thus making efficient use of the available resources, a problem in the study of underutilized species (Padulosi et al., 2002).

The themes considered in this proposal, of agricultural and alimentary interest to the population such as fruit quality, nutraceutical compounds, nutritional requirements for plant growing or phenological response in different environmental conditions from those where they develop, would facilitate their study.

The information generated would allow the selection of germplasm for direct use or for genetic improvement, and the identification of nutraceutical compounds or secondary metabolites with benefits for human health such as those reported in amaranth, moringa, buckwheat seeds or jaltomate (Jing et al., 2016; Platel, 2020; Nixwell et al., 2022; Flores-Sánchez et al., 2023).

In addition, the information generated would contribute to the promotion of the cultivation and consumption of these species, among low-income farmers in developing countries, where diversified agricultural systems are crucial to meet food needs in a sustainable way, and to the population in general by having food alternatives that benefit a more balanced diet. Furthermore, the production and commercialization of these alternative crops would serve to diversify the economic income of mainly low-income producers and actors linked to the value chain (Padulosi et al., 2013). All these issues contribute to biodiversity conservation and ecosystem resilience, since rescuing underutilized or abandoned plant species, wild, semi-domesticated or domesticated will preserve potential and alternative food sources.

With the application of this proposal, information could also be generated to contribute to the protection and floristic knowledge, where taxonomic studies based on morphological characteristics hinder the identification and classification of specimens that are difficult to distinguish morphologically, as reported in the genus *Jaltomata*, where a studied specimen was recognized as a new species for this genus, based on DNA sequencing studies and phylogenetic trees (Flores-Sánchez et al., 2024).

In this way, the study of these resources based on the proposed methodology could contribute to their conservation, protection and sustainable use.

## 6 Limitations of the proposal

Within the limitations of the proposal is the bias of the person interested in the study of these resources, since it will depend on the information he has and his criteria to prioritize the different aspects proposed for the selection of the species to study, which could lead to a waste of time and economic resources.

Also, there would be a limitation on access to sufficient economic resources and to the equipment and infrastructure required for the different aspects that are proposed to be studied.

Because it is a multidisciplinary study, another limitation is that the researcher has the knowledge of the different disciplines that are required, which would imply the investment of significant time and economic resources to acquire the knowledge and equipment necessary in these disciplines, therefore, the collaboration of other researchers interested in the study of these plant resources would be required.

The low availability of germplasm of some species to be studied would be another limitation for genetic improvement purposes, since it is necessary to have a wide genetic diversity of the germplasm studied that allows identifying important characteristics such as fruit size, stem diameter, nutritional content and nutraceutical compounds.

## 7 Conclusion

In this way, the proposal described seeks to contribute to the establishment of a methodology that facilitates the study and use of underutilized or abandoned species, through the generation of data that allows identifying germplasm for direct use or for the development of subsequent studies such as the identification of characteristics of agricultural interest for genetic improvement using molecular techniques that allow reducing times and economic costs in this process, the identification of secondary metabolites useful for humans and the cultivation conditions that affect their synthesis in a positive or negative way, or the cultivation conditions that allow obtaining products with adequate nutritional quality for people and, address the problem raised by the reduced diversity of foods available in production systems, making use of the available biodiversity, as raised by the World Health Organization and the Food and Agricultural Organization of the United Nations.
